# The Influence of Pelvic Ramus Fracture on the Stability of Fixed Pelvic Complex Fracture

**DOI:** 10.1155/2015/790575

**Published:** 2015-10-01

**Authors:** Jianyin Lei, Yue Zhang, Guiying Wu, Zhihua Wang, Xianhua Cai

**Affiliations:** ^1^Institute of Applied Mechanics and Biomedical Engineering, Taiyuan University of Technology, 79 Yingze Road, Taiyuan 030024, China; ^2^Mechanics College, Taiyuan University of Technology, Taiyuan 030024, China; ^3^Department of Orthopedics, Wuhan General Hospital of Guangzhou Command, 627 Wuluo Road, Wuhan 430070, China

## Abstract

This study aims to evaluate the biomechanical mechanism of pelvic ring injury for the stability of pelvis using the finite element (FE) method. Complex pelvic fracture (i.e., anterior column with posterior hemitransverse lesion) combined with pelvic ramus fracture was used to evaluate the biomechanics stability of the pelvis. Three FE fracture models (i.e., Dynamic Anterior Plate-Screw System for Quadrilateral Area (DAPSQ) for complex pelvic fracture with intact pubic ramus, DAPSQ for complex pelvic fracture with pubic ramus fracture, and DAPSQ for complex pelvic fracture with fixed pubic ramus fracture) were established to explore the biomechanics stability of the pelvis. The pubic ramus fracture leads to an unsymmetrical situation and an unstable situation of the pelvis. The fixed pubic ramus fracture did well in reducing the stress levels of the pelvic bone and fixation system, as well as displacement difference in the pubic symphysis, and it could change the unstable situation back to a certain extent. The pelvic ring integrity was the prerequisite of the pelvic stability and should be in a stable condition when the complex fracture is treated.

## 1. Introduction

The pubic symphysis, which includes the anterior pubic fibrocartilaginous disc, as well as anterior, posterior, inferior, and superior ligaments, connects the anterior portion of the two pelvic coxal bones as a nonsynovial joint [1]. Biomechanics analysis of the pelvis shows the inferior public ramus and superior public ramus functions as arches, which transfer the load in the lateral direction from one side to the other side and transfer the weight of the upright trunk from the sacrum to the hips [2]. The pubis symphysis and its surrounding ligaments (superior and inferior pubic ligament) connect these two load-bearing arches and maintain the mechanical integrity. The function of the pubic symphysis is to maintain the structural integrity of the pelvis and to provide joint stability by neutralizing shear and tensile stresses.

Minimally displaced pubic rami fractures are frequently seen at the emergency department after trivial accidents, especially among the elderly population. Pubic ramus fractures, which typically occur as lateral compression fractures after direct impact on the side of the lesion [3], are estimated to account for two-thirds of osteoporotic pelvic fractures [4]. The isolated pubic ramus fractures are low-energy fractures, and they are often considered to be relatively harmless and are typically treated in a nonoperative way.

Although complex or single fractures of the acetabulum combined with pelvic ring injury account for a small proportion of pelvic fractures, this kind of fracture varies in severity and requires a complicated procedure for it to be managed [5]. This fracture not only is a posttraumatic, high-energy periprosthetic fracture, but also involves a combined unstable pelvic fracture with a complex acetabular fracture component [5]. Complex fractures of the acetabulum or isolated pubic ramus fractures are largely underreported in literature, whereas (isolated) the pubic rami fractures are not specifically addressed, because pubic ramus fractures typically heal uneventfully. In addition, sporadic cases have been reported regarding the management of complex fractures in the acetabulum combined with pubic ramus fractures.

The biomechanics of the pelvis or its fractures are not yet thoroughly understood because of its complex geometry and structure. Therefore, performing a detailed study of its functional performance is helpful. Moreover, the pelvis is sensitive to fractures and disruptions of the pubic ramus. Several alternative methods have been used to study pelvic biomechanics, such as “in vivo” strain gages [6–9], photoelastic models [10], and FE analyses [11]. For the cadaveric study, it is still important source of the pelvic biomechanical. The veracity of the cadaveric study was restricted to the sample size and the cost of test. FE analysis, which is suited for parameter studies and determines more values than cadaveric studies, has been used to study the pelvis response to obtain in-depth insights on the biomechanics stability of the pelvis.

This study aims to explore the biomechanics stability of the pelvis with a complex fracture combined with pelvic ramus fracture via FE analysis. Three different models were used to appraise pelvic stability. The mechanism was evaluated based on the stress and displacement distribution and force transformation of the three models.

## 2. Materials and Methods

### 2.1. FE Model of Pelvis

**Figure 1:**
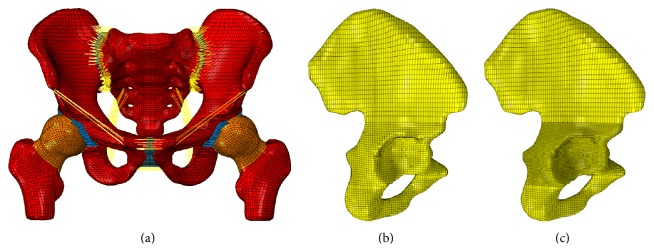
FE model of the pelvis and pelvic bone with different mesh length. (a) FE model of the pelvis; (b) iliac bone with large mesh length; (c) pelvic bone with small mesh length.

The CT scan images were obtained from Wuhan General Hospital of Guangzhou Command. The Hospital Ethics Committee licensed this study. Laser topography was conducted to create the pelvic model by using a 16-slice spiral CT with an accuracy of 0.5 mm (40 years old, 175 cm height, 65 kg weight). Bony tissues were meshed using a combined artificial and automatic division method in the software of ANSYS ICEM CFD 14.5 and Hypermesh 12.0. The cortical bone has a thickness of 1.5 mm according to previous studies [12, 13]. The soft tissues (i.e., end-plates, cartilage, pubic symphysis, and acetabular fossa) between bony tissues were automatically generated into hexahedral mesh grids in Hypermesh. In order to ensure the convergence of optimization and the consistence of displacement between adjacent tissues, shared nodes contact has been used between tissues in the software of Hypermesh. It is difficult to assign different materiel properties to different tissues in a whole model. Therefore, different pelvic tissues were assigned to single part. Tied contacts were used between tissues with surfaces that were adjacent to each other in the software of ABAQUS 12.0 to ensure that no relative displacement occurs. The main pelvic ligaments were dragged in Hypermesh and modeled into truss elements with a length of 2 mm. Furthermore, the material properties of the model were assumed to be homogeneous and isotropic [14–16]. In order to explore mesh sensitivity for the mechanical properties of pelvic, the acetabulum grids were refined from 2 mm into 1 mm in the acetabular bone. The FE model of the pelvis and the iliac bone with different mesh length is shown in Figure 1. The properties of pelvic bone and ligaments are shown in Tables 1 and 2, respectively.

### 2.2. Fragment Models and Surgical Techniques

**Figure 2:**
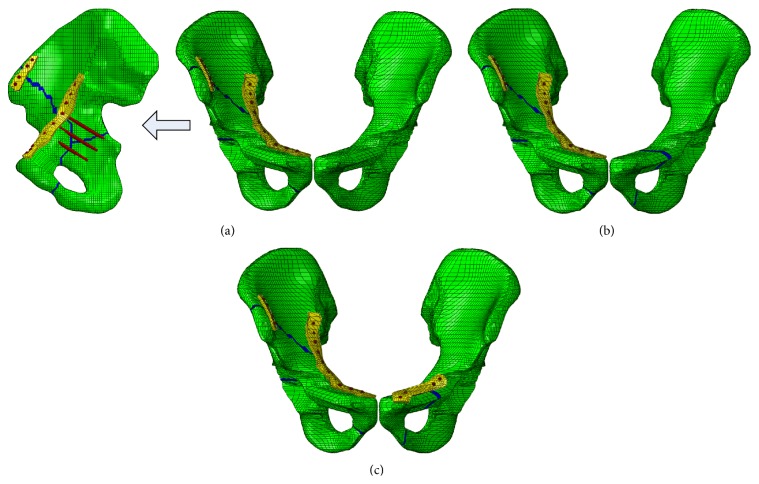
The FE model of the pelvis at different conditions. (a) The first model; (b) the second model; (c) the third model.

Anterior column with posterior hemitransverse lesion combined with pelvic ramus fracture is a relatively common fracture in car crash accidents. Letournel [17] showed that the anterior column with posterior hemitransverse lesion was determined using two converging lines, which originated from the anterior superior spine and ischial spine or just above the part, and these two lines merged in the center of the acetabular bone. Most commonly, this fracture type exists below the anterior inferior iliac spine or extends from the middle of the pubic ramus to any point above the anterior segment of the iliac crest (Figure 2(a)) [18]. These data constituted the anterior column with posterior hemitransverse lesion model. Pelvic ring injury almost constitutes the fracture of the superior pubic ramus (Figure 2(b)). The pelvis was unable to keep a stable condition when the fracture occurred. Series of mesh along the fracture line were removed to represent the fractures. The width of fracture gap was determined by the length of the mesh (2 mm in his paper). Additionally, the intercalary osteochondral fragments became free after fracture occurred, but had little or no effect on supporting the body weight. It has been suggested that the stabilization of a fracture is mainly a process of the cancellous bone rather than a process of the cortex, with fractured bone presenting the same morphology of callus as described for microcallus formations [19, 20]. In this case, the elasticity modulus weakened to 1/10 of the normal bone means the fracture line was weakened to 15 MPa [19, 20].

The fragments were unable to keep the original position because of bone fracture; thus, the fixation system was added to return the acetabular into a stable state. The primary approach for complex pelvic fracture was to achieve a bony union through fracture reduction while maintaining the original fracture components as well as preserving bone stock for future reconstruction, if necessary [5]. The treatment principle for pelvic fractures, including pelvic ring fractures, should be based on anatomic reduction and easy rigid fixation [21, 22]. The complex pelvic fractures can be treated through open reduction and internal fixation, which often consists of reconstructing plates and lag or interfracture screws.

The pelvic reduction was unstable when the pubic ramus fracture occurred. Open or closed reduction and early internal or external fixation of the pubic ramus allow healing with no residual deformity. The pubic symphysis was finally reduced by means of superior pubic ramus osteotomy to unlock the incarcerated pubic body out of the contralateral obturator foramen. A reconstruction plate was contoured to the super pubic symphysis, which was fixed to the central fragment.

The anterior column with posterior hemitransverse lesion was built on the right side hemipelvis and the pubic rami fractures on the contralateral side. DAPSQ was adopted for the complex acetabulum fractures. Placing lag screws via an anterior approach is a novel method to cure complex acetabular fractures [15, 23], which has obtained a China state patent [24]. The lag screws that screwed through the anterior column to the posterior column could produce a particular clinical result, but its fixation is eccentric (partial posterior) and requires the fracture blocks of the anterior and posterior columns to not be crushed.

The plates and screws were made of the Nitinol (NiTi) shape-memory alloy because of their inherent advantages (i.e., shape-memory effect, remarkable resistance to wear and corrosion, and good histocompatibility) [25]. The elasticity modulus and the Poisson ratio of the NiTi shape-memory alloy were 110 GPa and 0.3, respectively. The contact between plates and cortical surface was defined as face-to-face contact with a friction coefficient of 0.1. Coupling constraints were used between plates and screws in order to make sure no relative sliding occurs. The screws were embedded in the pelvic bone, which are used to specify an element or a group of screws or elements that lie embedded in the pelvic whose response will be used to constrain the translational degrees of freedom of the screws nodes [26].

To analyze the influence of the pubic ramus, three models were created as follows:

 The first model: DAPSQ for anterior column with posterior hemitransverse lesion on right side, which is combined with pubic bone integrity on the contralateral side (Figure 2(a)).
 The second model: the same condition was used in the right side, whereas the superior and inferior pubic ramus were ruptured without a fixation system (Figure 2(b)).
 The third model: The titanium plate fixation system was settled for the pubic ramus fractures (Figure 2(c)).

### 2.3. Loading and Boundary Conditions

The double-limb stance was exerted on each model. The physiological load was similar to existing models, as described in Sawaguchi et al. [27]. The model was placed in a specific neutral position that was defined with the iliac wings level (coplanar in the horizontal plane) [28]. In the sagittal plane, the proximal femoral shaft was vertical. The degrees of freedom at the end of the femur were restrained to represent the double-limb stance. The body weight of 600 N was loaded on the upper surface of the sacrum.

**Figure 3:**
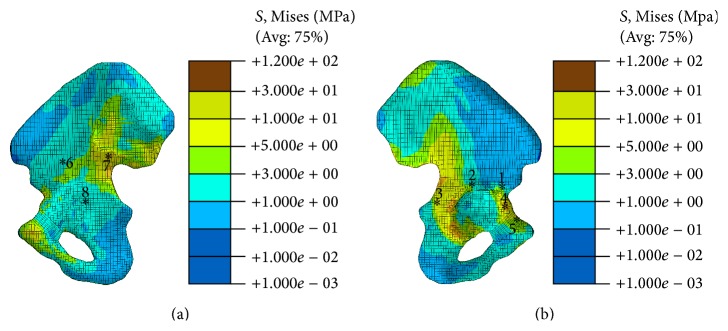
Stress distribution in the pelvic bone and the location of gages. (a) Medial view of the stress in pelvic bone; (b) lateral view of the stress in pelvic bone.

*Validation of the Pelvic Model and Fixation System: DAPSQ*. Medial and lateral views of the von Mises stresses that were observed in the cortical bone of pelvic bone are shown in Figure 3. In terms of the von Mises stress level, the present models and the existing model [12, 15] or in vivo experiments data [29] were in agreement. The regions of stress concentration were observed at the superior rim of the acetabulum and on the ilium superior to the acetabulum. In order to validate the FE model, eight points (which were corresponding to position in the Dalstra vivo experiments [29]) were chosen to evaluate the von Mises stress level. The average von Mises stress in eight positions was 2.68 MPa, which was slightly larger than the value obtained though the Phillips simulation (about 2 MPa) and Dalstra vivo experiment (1.73 MPa in the left pelvic bone and 2.02 MPa in right side). The difference may be due to the ignorance of the sacrum or the femur, or the difference of the loading and boundary conditions (the force was loaded though acetabulum).

**Figure 4:**
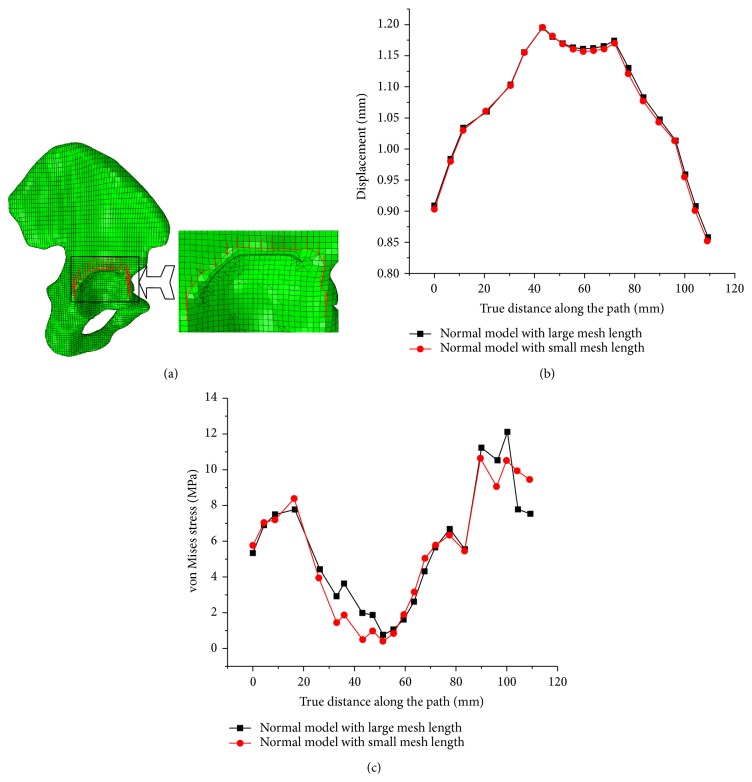
The path across the limbus of acetabulum and its displacement and stress distribution along the path. (a) Path across the limbus of acetabulum; (b) displacement disrtribution along the path; (c) von Mises stress distribution along the path.

In order to explore mesh sensitivity for the mechanical properties of pelvic, a path across the limbus of acetabulum was generated (Figure 4) to evaluate mesh sensitivity for stress and displacement distribution. The stress and displacement distributions in two FE models with different mesh size were almost the same. Therefore, mesh sensitivity studies revealed that further refinement does not significantly improve calculation accuracy. So, the findings all show that the FE model developed in this study produces stress field which was similar to those reported in previous literatures, and could meet our needs [12, 15].

Acetabular fractures are commonly treated using reconstruction plates and fixation or lag screws. Moreover, the reconstruction plate could effectively buttress the fracture fragments to keep the fracture component in the original position. The screws could fit closely to the irregular surfaces to overcome the resistance generated from shear and torsion. Meanwhile, quadrilateral area screws that pass through the anterior column to the posterior column could generate a good therapeutic effect because the quadrilateral screws were fully inserted into the cortical bone surface to generate higher stiffness than fixation screws, which were only inserted into two ends.

**Figure 5:**
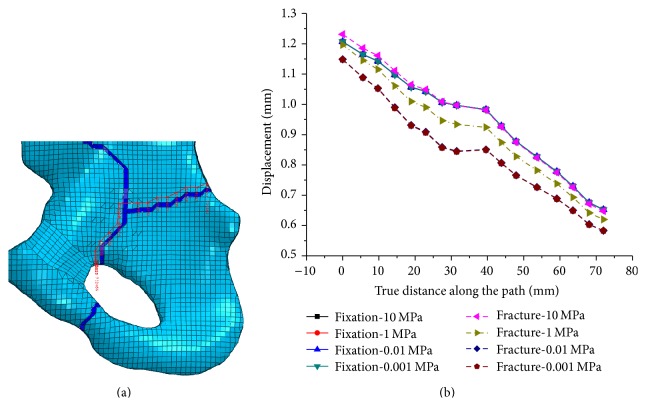
The path along the upper fracture line and its displacement distribution along the path with different elasticity modulus. (a) The path along the upper fracture line; (b) displacement along the path with different fracture line modulus under different situation.

Additionally, the elasticity modulus of the fracture line has little effect on the stability of the pelvic. A path along the upper fracture line was generated (Figure 5(a)). The displacement along the path under different elasticity modulus of fracture was shown in Figure 5(b). Four different values of elasticity modulus (0.01, 0.01, 1, and 10 MPa) were assigned to fracture line. There is a great deal of difference between these values of the displacement along the path. while once the fixation system (DAPSQ) applied, the difference reduced to the minimum size. Therefore, the value of elasticity modulus of fracture line has little effect on the stability of the pelvic.

## 3. Results

**Figure 6:**
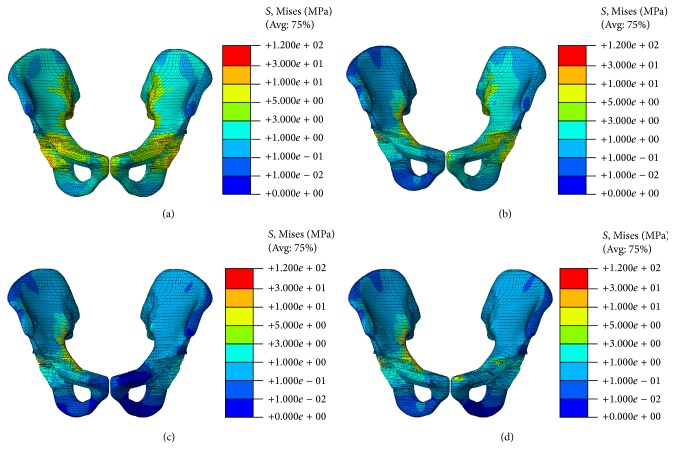
Stress distribution at different conditions. (a) Nonfracture model; (b) the first model; (c) the second model; (d) the third model.

The stress distribution of the pelvic bone is shown in Figure 4. In the nonfracture model, the stress value in superior ramus of pubis was approximately 4 MPa, which is almost the same across the acetabulum [15]. Therefore, the pelvic ring has an important function in transferring axial force from the upper body to the lower limbs. In the first model (DAPSQ combined with intact contralateral pubic ramus), the intact pubic ramus suffer larger stress and transfer larger force than the nonfracture model (Figure 6(b)). The force transformation was blocked when the fracture occurred, and the force could not be transmitted from the pubic ramus to the pubic symphysis (Figure 6(c)). Meanwhile, the force could transfer through the fixation plates but cannot return to its intact state when the fractured ramus was fixed (Figure 6(d)), whereas the pubic tubercle which adhered to the pubic symphysis suffers considerable stress (Figure 6(d)).

**Figure 7:**
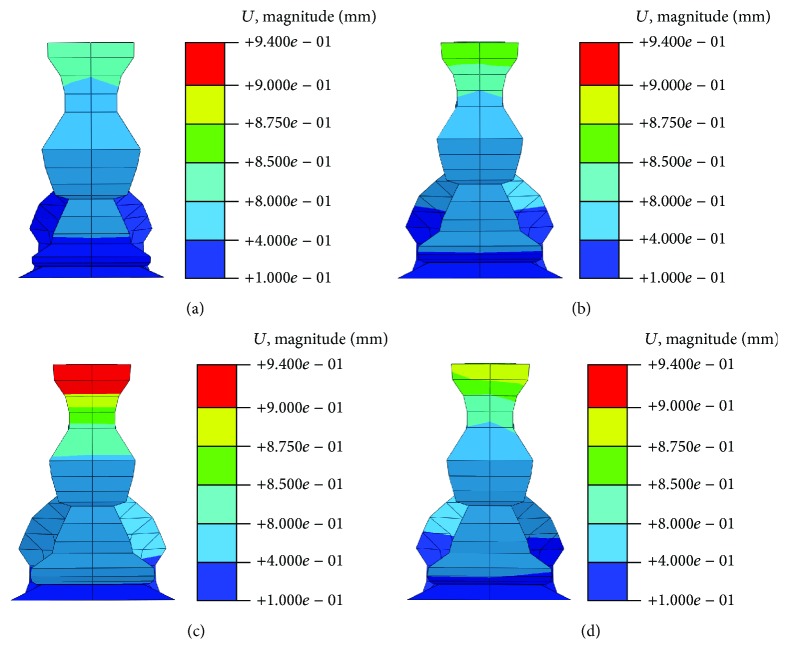
The nodal resultant displacement distribution of pubic symphysis at different conditions. (a) Nonfracture model; (b) the first model; (c) the second model; (d) the third model.

The pubic symphysis is a nonsynovial amphiarthrodial joint, connecting two pubic bones. The resultant displacement distribution is shown in Figure 7. All three fracture models could not match the nonfracture model; thus, the pelvic hardly returns to normal conditions after the fractures, although the DAPSQ could reduce the maximum displacement of pubic symphysis. The nodal displacement of the pubic symphysis was not consistent on both sides in all three fracture models, especially in the second model. In the first and third model, the larger displacement occurred in the right pelvic side (the DAPQS side). The complex pelvic fractures were more destructive than pubic ramus fracture although fixation system was applied, while, in the second model, the largest displacement was shown in the left pelvic side. The maximum displacement of the pubic symphysis in all models occurred in the second model (complex pelvic fracture combined with pubic ramus fracture). The fractured pelvic under the second condition was in insufficient conditions because the pelvic has a large displacement and in an asymmetric situation. Thus, the pelvis that suffered complex fracture combined with pelvic ramus fracture was unable to remain in a stable condition. The displacement level for the third condition ranged between the two former values. Therefore, pelvic ring integrity was the prerequisite of pelvic stability.

**Figure 8:**
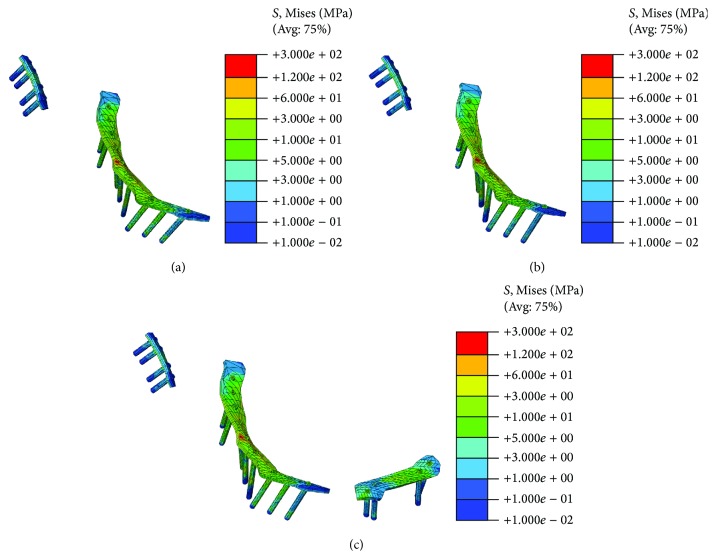
Stress distribution of fixation systems at different conditions. (a) Nonfracture model; (b) the first model; (c) the second model; (d) the third model.

The stress distribution of the fixation system was shown in Figure 8. The DAPSQ in the second system (Figure 8(b)) suffers from higher stress than the other two conditions. The highest stress, which was larger than 120 MPa, was observed in the center of the reconstruction plate at the same condition. The stress distribution pattern in the DAPSQ combined with fixed pubic ramus fracture (Figure 8(c)) fell between the former two conditions. Therefore, pelvic ring integrity or stability was needed when the pelvis suffers from a complex pelvic fracture.

## 4. Discussion

FE analyses of the pelvic stability for fracture are rare because of the complex three-dimensional geometry, the difference of fracture types, and the controversy of fixation systems. This study aims to explore the biomechanics stability of pelvis with complex fracture combined with pelvic ring injury through FE analysis. A complete, accurate, and validated pelvis model was developed, and complex pelvic fracture combined with pelvic ramus fracture was created to represent unstable condition. Three different models were used to appraise the pelvic stability mechanism. The mechanism was evaluated based on stress and displacement distribution and force transformation of the three models.

The study was based on FE analysis; thus, the following points should be noted. Firstly, the accuracy of the pelvic model affects the veracity of the FE result. For a real pelvis, relatively high values for the thickness of cortical bone were allocated at particular locations, and creating a pelvic model to match the real pelvis model was difficult [12]. However, previous studies have shown that cortical bone stress was not sensitive to changes in cortical thickness [12]. In addition, the pelvis ligaments were modeled using truss elements with elastic modulus because no 3D geometric models of the pelvis ligaments were available. The elastic approximation is accurate enough for a comparative study of pelvis stability [30]. Secondly, finding a universal fixation system for the complex fracture combined with pelvic ring injury is impossible because the fracture varies in severity and diversity, which needs a complex process for orthopedist to conduct operation [15, 23]. Moreover, specific problems for fixation systems, such as heterotopic ossification, abductor weakness, and slipping out or breakage of the fixation system, should be a concern in the clinical results. Therefore, a large-scale, formal study should be conducted in the future to enhance the precision of our conclusion.

The symphysis pubis is a nonsynovial amphiarthrodial joint that forms a fibrocartilaginous union between the two pubic bones [2]. This articulation often falls outside the mainstream interest of orthopedic surgeons because dramatic symptoms or signs are seldom produced. And the operation for isolated pubic ramus fracture was not necessary. However, pelvic ring integrity plays a pivotal role in keeping the stability of pelvis [31]. Four injury patterns are apparent at the symphysis pubis: diastases, straddle fractures, intraarticular fractures, and overlapping dislocations, as well as combination fracture-dislocations. Isolated pubic rami (diastases and straddle fractures) fractures are common fractures, and the treatment for these fractures is typically nonoperative. Patients with isolated pubic rami fractures have a good prognosis with regard to long-term pain relief and functional outcomes. Meanwhile, arthrodesis may be the only therapeutic option in severe and recalcitrant cases (i.e., overlapping dislocations or combination fracture-dislocations). The treatment for these cases must have a complicated process to complete, that is, an early and rapid reduction and fixation of massively displaced and unstable fractures.

Few scholars are involved in the general management of the treatment for complex fractures combined with pelvic ring injury. Open reduction and internal fixation is the general method for complex fracture [32], whereas a closed reduction of the diastasis and stabilization with external fixation provides an optimized therapeutic option for isolated pubic ramus. Complex pelvic fracture combined with pelvic ring injury varies in severity and diversity, and this kind of fracture can occur concomitantly with a complicated procedure to manage [5].

Analysis of the pelvic ring shows that the skinny regions function as arches that transfer the weight of the upright trunk from the sacrum to the hips and transfer the load in the lateral direction from one side to the other side [2]. The superior and inferior pubic ramus on each side formed two “little” arches to increase pelvic stiffness. The functions of the joint are to absorb shock during walking and allow the delivery of body weight. The stress distribution of the pelvic bone shows that a major part of the body weight was transferred though the pelvic ring, especially from the superior pubic ramus. These findings were consistent with those from previous studies [2]. Meanwhile, the stress level in the pubic rami of the nonfracture model was approximately 4 MPa (obtained by average the stress value along the pubic rami) compared with the fracture model that was less than 0.5 MPa. The force transformation pattern changes when the fracture occurred: the vertical force was mainly transferred through the acetabulum to the hips or though the fixation plates to the other side rather than through the fracture line. The fractured pubic ring contributed little to the pelvic stability.

The rank of the pubic symphysis displacement and its difference are as follows: the second model (DAPSQ combined with pelvic ramus fracture) possesses the maximum value, and the third model (fixed the pubic ramus) followed. The pelvic ramus fracture makes the pelvis suffer from large displacement and low rigidity, which leads to continuous unstable conditions of the pelvis, whereas the fixation of the pubic ramus fracture could change the fracture bone back to a particular extent. Therefore, pelvic ring integrity was the prerequisite of pelvic stability. The models also indicated that the symphysis was subject to a combination of superior/inferior glide and lateral compression under asymmetric structure by analyzing the displacement distribution [33]. Biomechanics analysis of the pelvis shows that the pubis symphysis and its surrounding ligaments could neutralize the shear and tensile stresses to provide joint stability and maintain the pelvis integrity.

The plate regions that attached to the fracture line or screws are more stressed than those in other positions; the screws are the same. These findings may be ascribed to either the difference in the relative displacements of the split parts or to the changeable in the material. These two reasons could generate shearing force or torque to produce a large damping on the fixation systems. Furthermore, the function of each part in the fixation system can be explained through the stress level, which means that the higher the stress the fixation system component suffers, the greater its function is. The maximum stress was observed in the reconstruction plate; thus, the plate had dominant function in maintaining the stability of the fracture model. The lag screws are more stressed than the screws fixing the plates, which could be attributed to the fact that the lag screws were fully inserted into the cortical bone surface to generate higher stiffness than fixation screws, which were only inserted into two ends.

FE models have been extensively used in evaluating the stability or the fracture of the pelvis [30, 34, 35]. These studies mainly focused on the influence of the direction, magnitude, or point of application of loading for the pelvis. However, this paper differs from previous works because we created a complex pelvic fracture combined with pelvic ring fracture, which was created under a clinician's guidance. Moreover, rather than applying force directly to pelvic bone or iliac fossa, the body weight in this paper was loaded on a complete pelvic model, which included bony structures with soft tissues (endplates and cartilage) and mainly pelvic ligamenta. The results, therefore, were more eloquent than those from simplistic models. Another novel approach of our study is that the traumatic biomechanics obtained from this paper could be used to guide surgical correction.

## 5. Conclusion

Complex pelvic fracture combined with pelvic ramus fracture was used to evaluate the biomechanics stability of the pelvis. The pubic ramus fracture leads to an unsymmetrical situation and an unstable situation of the pelvis. The fixed pubic ramus fracture did well in reducing the stress levels of the pelvic bone and fixation system and the displacement difference in the pubic symphysis and could change the unstable situation back to a certain extent. Therefore, the pelvic ring integrity was the prerequisite of the pelvic stability and should be in a stable condition when the complex fracture is treated.

## Figures and Tables

**Table 1:** Material properties of the pelvic bone [14–16].

Tissue	Elasticity modulus (MPa)	Poisson ratio (*ν*)	Thickness (mm)	Element number	Node number
Bone					
Cortical bone (sacrum)	17000	0.3	1.50	8752	17412
Cancellous bone (sacrum)	150	0.2		18524	22960
Cortical bone (ilium)	17000	0.3	1.50	7764∗2	14687∗2
Cancellous bone (ilium)	150	0.2		15809∗2	18628∗2
Cortical bone (femur)	17000	0.3	1.50	3151∗2	6304∗2
Cancellous bone (femur)	150	0.2		7856∗2	9520∗2
Soft tissues					
End-plate (sacrum)	24	0.4	0.23	1493∗2	1098∗2
Cartilage (sacrum)	54	0.4	1.81	468∗2	1036∗2
Cartilage (ilium)	54	0.4	0.80	468∗2	1036∗2
End-plate (ilium)	24	0.4	0.36	483∗2	1036∗2
Pubic symphysis	5	0.495		246	396

**Table 2:** Material properties of the pelvic ligaments [13].

Tissue	Ligament length (mm)	Attached area (mm^2^)	Elasticity modulus (MPa)	Poisson ratio (*ν*)
Sacroiliac ligament ring	14	1391	350	0.495
Sacrospinous	52	112	29	0.495
Sacrotuberous	90	539	33	0.495
Inguinal	96	45	2.6	0.495
Superior pubic	27	97	19	0.495
Arcuate pubic	25	156	20	0.495

## Abbreviation

DAPSQ: Dynamic Anterior Plate-Screw System for Quadrilateral Area.
